# Acetic Acid Bacteria and the Production and Quality of Wine Vinegar

**DOI:** 10.1155/2014/394671

**Published:** 2014-01-21

**Authors:** Albert Mas, María Jesús Torija, María del Carmen García-Parrilla, Ana María Troncoso

**Affiliations:** ^1^Facultad de Enología, Universitat Rovira i Virgili, Marcel*·*lí Domingo s/n, 43003 Tarragona, Spain; ^2^Facultad de Farmacia, Universidad de Sevilla, Profesor García González 2, 41012 Sevilla, Spain

## Abstract

The production of vinegar depends on an oxidation process that is mainly performed by acetic acid bacteria. Despite the different methods of vinegar production (more or less designated as either “fast” or “traditional”), the use of pure starter cultures remains far from being a reality. Uncontrolled mixed cultures are normally used, but this review proposes the use of controlled mixed cultures. The acetic acid bacteria species determine the quality of vinegar, although the final quality is a combined result of technological process, wood contact, and aging. This discussion centers on wine vinegar and evaluates the effects of these different processes on its chemical and sensory properties.

## 1. Introduction

Vinegar production dates back at least to 200 BC, and it is an illustrative example of microbial biotransformation. However, vinegar has always been seen as a “leftover” in the family of fermented products [1]. Vinegar has been part of the human diet as a condiment and food preservative, as well as the basis for simple remedies for people and animals, since remote antiquity. However, its production was always considered a chemical process. As mentioned in the review on vinegar history [1], in 1732, the Dutchman Boerhaave noted that the “mother of vinegar” was a living organism, although he did not specify the role of this organism in the process of acidification. We shall refer to this process as “acetification” instead of the more popular “acetous fermentation” due to its strict requirement for oxygen. Lavoisier in 1789 demonstrated that acetification is the oxidation of ethanol, but he did not suspect a role for living organisms. Persoon in 1822 described the film formed at the surface of wine, beer, or pickled vegetables and the biological nature of such substances, and in “European Mycology,” he added new species of *Mycoderma*: *ollare*, *mesentericum*, *lagenoe,* and *pergameneum*. Chaptal also observed that the production of vinegar went well when the “wine flower”, whose appearance heralds and precedes the acidification, appeared on the surface of the wine. However, Berzelius warned that all decaying organic matter developed the same type of flora if it was exposed to air. Acetification became part of the controversy between scientists such as Berzelius and Liebig; some argued that the process was purely chemical, and some claimed that this transformation involved an “organized living being.” Regarding the “mother of vinegar,” Kützing noted in 1837 that the thin film that covered the surface of the liquid was made by “globulles” six times smaller than yeasts; thus, he can be credited with the first microscopic observation of acetic acid bacteria in 1837. Finally, Pasteur in 1864 claimed that the transformation of wine into vinegar was due to the development of the veil of *Mycoderma aceti* on its surface [1].

Despite some small local differences, in general, food regulations consider vinegar to be the result of a double fermentation (alcoholic and acetous or acetification) of any sugar substrate. European countries have specific rules for vinegars sold in different regions. In the European Union, the established limits for acidity and residual ethanol content are strictly set. Thus, the acidity of wine vinegar (acetification obtained exclusively from wine) must be at least 6% (w/v), and the maximum residual ethanol allowed is 1.5% (v/v). However, the variety of raw materials used in the production of vinegar is very great, ranging from byproducts and agricultural surpluses to high-quality substrates for the most unique and prized vinegars, such as Sherry vinegar (Spain) and Aceto Balsamico Tradizionalle (Italy). The quality standard defines up to ten types of vinegars, which include wine vinegar, fruit, cider, alcoholic, cereal, malt, malt distillate, balsamic (with added grape must), and “other balsamic vinegars,” which encompass any other substrate of agricultural origin, such as honey or rice. Undoubtedly, wine vinegar is the most common type in Mediterranean countries, although the latest gastronomic trends have led to a considerable expansion of the varieties available in recent years. However, worldwide most of the vinegar produced is “white” vinegar, that is, vinegar produced directly from diluted alcohol. In this review, we will focus mostly on wine vinegar and the role of acetic acid bacteria and the quality of this product.

## 2. Production Technology of Wine Vinegars

Apart from their different substrates, vinegars can also be differentiated by their production systems. In traditional vinegars, the transformation of ethanol into acetic acid is performed by a static culture of acetic acid bacteria at the interface between the liquid and air. The barrels are filled to 2/3 capacity to leave an air chamber, which is kept in contact with the outside air using one of various types of openings. This production system is called “surface culture,” and this process is considered the traditional method. The more standardized version of this method, the “Orleans method,” includes side holes for air circulation and adds a funnel with an extension to the base of the barrel to allow wine to be added at the bottom of the barrel, preventing the alteration of the “mother of vinegar,” that is, the biofilm formed by acetic acid bacteria on the surface. The vinegars produced by this traditional system are generally considered of high quality because of their organoleptic complexity. In fact, the product quality results from (i) the raw material (wine or other substrate), (ii) the metabolism of the acetic acid bacteria, which produce some additional transformations (mostly oxidation reactions, but also ester formations, e.g.) on top of the basic transformation (ethanol to acetic acid), (iii) the interaction between the vinegar and the wood from the barrels, and (iv) the aging process, which integrates all of the previously mentioned characteristics. However, the characterization of wine vinegar as a byproduct means that its production is often inadequately performed and includes many unnecessary risks. The groups participating in this review, together with the group of the University of Modena and Reggio Emilia and the University of Geneve, in collaboration with 3 vinegar companies (Acetaia Cavalli of Reggio Emilia, Italy, Viticultors Mas den Gil, from Priorat, Spain, and Vianigrerie ala Guinelle, from Banyuls, France) and one barrel making company (Boteria Torner, Penedes, Spain) developed the EU WINEGAR Project (wood solutions for excessive acetification length in traditional vinegar production 6th Framework Program). The WINEGAR project aimed to find alternative methods to improve and shorten the process without compromising the quality of the end product. The project focused on changes in a number of parameters: (i) the raw material used; (ii) the use of barrels specially designed for the development of the product, including assessment of wood type, barrel shape, volume, and use of new wood; and (iii) the selection of acetic acid bacteria starter cultures. The combination of these changes significantly sped up the process (a process that originally took between six months to a year was reduced to 50 days [2]) and maintained or increased the sensory quality of the product [3, 4].

However, there are also other methods that have been used to reduce the acidification time, such as the Schutzenbach system or systems with submerged cultures. In the first type, the bacteria are immobilized on wood chips, forming a solid bed on which the vinegar spreads. After this vinegar passes through the bed of chips, it is collected in a container at the bottom and pumped back to the same fixed bed. The acidity successively increases, and it is possible to obtain vinegar of reasonable quality within a week.

Submerged culture systems provide a much faster alternative. These systems rely on suitable turbines to generate a flow of air bubbles into the wine or alcoholic solution. The oxidative process occurs in the air-liquid interfaces of the air bubbles. Improvements to this process generally involve engineering (maintenance and persistence of the bubbles in the liquid, uniformity of the bubble size, recovery of lost aromas, etc.). In this type of vinegar, the bacteria become bioreactors for the transformation of alcohol into acetic acid, with only very limited production of other metabolites. The airflow also contributes to considerable loss of the volatile compounds present in the original wine, resulting in more organoleptically limited product that was produced at a significantly lower cost. Although early containers for submerged culture processing were made of wood, the most current containers are stainless steel, which is more hygienic and resistant to wear. Although the wood containers were meant to provide some organoleptic complexity, there was hardly any transfer from the wood to the vinegar because of the imbalance between contact surface and volume and the speed of the process. This limitation can be compensated for by subsequent aging in barrels or incubation with wood fragments or wood chips, which may contribute to the recovery of some missing organoleptic characters. Despite the loss in product quality, this methodology has two important advantages: speed (the vinegar is produced in cycles of 24 hours) and acidity (the product can reach concentrations of acetic acid of up to 23–25%, compared to 6–13% achieved with other systems). Higher acidity helps to reduce transportation costs by reducing water transport.

An important aspect that contributes to the organoleptic quality of vinegars is aging. In fact, this is a fundamental aspect of the integration of the different compounds in vinegars. The increase in organoleptic quality after aging is remarkable; in addition to interactions with the wood, a series of chemical reactions, evaporation, the production of esters, reactions between acids and residual alcohols, and other processes result in better integration of aromas and metabolites and a reduction in the pungency of acetic acid.

## 3. Acetic Acid Bacteria

Although acetic acid bacteria are feared among oenologists because of their negative effects on grapes and on wine in general, they are the main agents in the production of vinegar. Acetic acid bacteria are Gram negative or Gram variable, ellipsoidal or cylindrical, and can be observed under the microscope alone, in pairs or in aggregates and chains [5]. Acetic acid bacteria have aerobic respiratory metabolism, and oxygen is generally used as the final electron acceptor; however, other compounds may occasionally act as final electron acceptors, allowing the bacteria to survive under nearly anaerobic conditions, such as the ones present during wine fermentation [6]. The bacteria's growth in these media is severely limited, and they may remain viable but not culturable [7]. These bacteria are found on substrates containing sugars and/or alcohol, such as fruit juice, wine, cider, beer, and vinegar. On these substrates, the sugars and alcohols are incompletely oxidized, leading to the accumulation of organic acids, such as the production of acetic acid from ethanol or gluconic acid from glucose. Some of the transformations performed by acetic acid bacteria are of great interest to the biotechnology industry. Despite this interest, the role of these bacteria in vinegar production remains their most familiar and extensively used industrial application.

The metabolism of some acetic acid bacteria may include a tricarboxylic acid cycle function, enabling them to completely transform acetic acid to CO_2_ and water [5]. However, because entry into the acetate cycle is inhibited by the presence of ethanol, it is essential to maintain a low concentration of ethanol in the presence of acetic acid bacteria to prevent this full oxidation. In fact, ethanol concentrations between 0.5 and 1% are regularly maintained in vinegars.

Acetic acid bacteria have been considered “fastidious” due to their response to growth in culture media. Their cultivability is often lower and more irregular than that observed under the microscope, and these differences can be of several log units [8]. Many strains lose some features (e.g., the ability to produce appreciable concentrations of acetic acid) after growth in culture media. Species identification has traditionally been performed by physiological and biochemical tests, and only half a dozen species from the genera *Acetobacter* and *Gluconobacter* were identified. These two genera could be differentiated based on their preference for alcohol or glucose as a substrate [5]. However, the use of molecular methods has improved efforts at taxonomy, and there are currently 14 genera and approximately 70 species described [9]. Approximately one dozen species and more than 40 strains have been sequenced. Some of the best-known species in the production of vinegars have been transferred from different genera. For example, three of the oldest species described in the production of vinegar were initially classified as genus *Acetobacter,* reclassified as *Gluconacetobacter* [10], and more recently moved to *Komagataeibacter* [9]. These species, *Komagataeibacter europaeus, hansenii, *and* xylinus*, now appear in the literature or textbooks under three different genera.

These molecular methods and their adaptation to the conditions of routine studies for the analysis of populations and the control of microbiological processes have been studied by a group at the Rovira i Virgili University. We have developed a number of methods for the routine identification of species by restriction analysis of ribosomal genes or their spacers [11, 12], which has allowed us to better understand the process of appearance and resistance during the alcoholic fermentation and vinegar production process. Likewise, we applied methods for strain-level identification, which allowed us to track acetic acid bacterial populations from grape to wine and during the process of vinegar making [13, 14]. However, we routinely applied these methods for the analysis of populations recovered in culture media, which has the disadvantage of low recovery, as mentioned above. In recent years, other molecular applications have allowed us to use independent methodologies such as DGGE culture [15–18] or quantitative PCR [8, 19–21]. These methods provide us with additional opportunities to follow the acetic acid bacterial populations in wine or vinegar.

Focusing on the production of wine vinegar, the use of these techniques has allowed us to observe that the vinegar is produced by a succession of strains and species, depending on the concentration of acetic acid [22]. At low concentrations of acetic acid, species of the genus *Acetobacter* predominate. *A. pasteurianus* seems to be the most common in wine vinegars, although other *Acetobacter,* such as *A. malorum*, *A. cerevisiae,* or *A. aceti,* may also be frequent in other fruit vinegars [14, 23]. However, when acetic acid concentrations exceed 5%, the species from the former *Gluconacetobacter* take over the process, with species such as *Komagataeibacter europaeus* or *Gluconacetobacter intermedius* predominating. This transition has also been observed in processes where we inoculated pure starter cultures of *Acetobacter pasteurianus,* during the WINEGAR project. In these cases, we observed that the starter cultures of *A. pasteurianus* effectively initiated the process but were later replaced by *Komagataeibacter europaeus *[24, 25]. This can be explained by both the differing acetic acid tolerances of the species and the presence of a contaminating population of acetic acid bacteria in the raw material (wine). At present, we believe that the best controlled process should include a starter formed by a mixed culture of a “quick start” acetic acid bacterium (*A. pasteurianus* or similar) and another with a high tolerance to acetic acid (*Komagataeibacter europaeus* or similar) to guarantee the best vinegar production through a rapid start and a good ending for the process.

## 4. Chemical Composition and Quality of Vinegars

The final quality of vinegars depends on the selection of appropriate starter cultures (generally mixed cultures) to lead the process. However, other factors include the quality of the starting material, the production method, and, if applicable, aging. In general, it is relatively easy to appreciate the sensory differences between products made by traditional methods and those manufactured on an industrial scale. Thorough characterization and quality evaluation requires the determination of the content of a number of compounds and sensory analysis. In recent years, there have been significant advances in the elucidation of the compounds responsible for the sensory quality of the products, and production methods have been changed to obtain vinegars with high acceptance at very competitive prices. The group at the University of Sevilla has been focusing on the characterization of wine vinegars for the last 20 years.

Aromatic compounds have a decisive effect on the quality of vinegars. The aroma is a complex fraction, containing many compounds with a wide range of volatilities, polarities, and concentrations ranging from several mg/L to ng/L. To date, we have identified more than 100 different chemical compounds in the aroma of wine vinegar, including carbonyl compounds, ethers, acetals, lactones, acids, alcohols, phenols, and volatile esters, all of which are involved to different extents in the final flavor [4]. During the aging process, the contact with wood produces a substantial increase in the aromatic complexity [26]. However, not all volatile compounds are responsible for the aroma of the product. They must not only reach odorant receptors but also interact with them in the olfactory epithelium, and not all volatile compounds are active odorants. The use of techniques based on gas chromatography coupled with olfactometry has allowed the contribution of each volatile compound in the final vinegar to be evaluated. For example, it has been determined that the characteristic aroma of Sherry vinegar involves several volatile compounds, such as diacetyl, isoamyl acetate, isovaleric acid, ethyl acetate, and sotolon [27].

Polyphenolic compounds, which are ubiquitous in plant products, are of great interest as quality determinants because, in addition to their antioxidant activity, they are responsible for the color and astringency of vinegar. Acetification is an aerobic process, and oxygen is critical to the growth of the bacteria. The reactivity of phenolic compounds and oxygen is specifically analyzed in winemaking for its relationship to the browning of white wines and the reactions of anthocyanins in red wines. The rate of acetification is also expected to be related to the solubility of oxygen in the medium, a decisive factor in the phenolic composition that can be useful for determining the method by which vinegar is produced. It should be emphasized that submerged systems use excess oxygen to secure and accelerate the process, whereas oxygen availability is limited in superficial cultures because it is continuously taken up by acetic acid bacteria. Additionally, oxygen affects the classes of polyphenolic compounds to different degrees. For example, the flavonol content of vinegars is largely influenced by oxygen availability during submerged fermentation. In contrast, surface acetification vinegars do not affect phenolic aldehydes, which are released from wooden barrels into the product [28].

The evolution of phenolic compounds during acetification in submerged culture systems has been studied in both laboratory and industrial fermenters. In a laboratory fermenter with Sherry wine as the substrate, the phenolic profile was not significantly altered [29]. However, a 50% decrease in phenolic compounds, mainly anthocyanins, has been reported in red wine vinegar [30].

The aging process involves the reaction of compounds over time: both the polymerization and release of compounds from the wood and losses through evaporation. The substances provided by the wood will depend on the type of wood and roasting, the ratio of the contact surface to liquid volume, and the aging time. As a consequence, significant differences have been observed in the phenolic composition of Sherry vinegars aged two or more years in static or traditional solera systems [31]. An observation of the evolution of phenolic compounds in Sherry vinegar aged in oak barrels showed that there were significant differences in the compounds vanillin, syringaldehyde, coniferyl aldehyde, and cinnamic acid after 90 days of aging [32]. Indeed, a 100% correct classification of vinegars aged for different periods of time was achieved by means of linear discriminant analysis using phenolic aldehydes as the variables. Furthermore, certain flavonoids are chemical markers of the wood that the vinegar has been in contact with; (+)-dihydrorobinetin is a characteristic compound released from nontoasted acacia wood, while (−)-taxifolin is typically released from cherry wood [33].

However, the consumer's perception of the product is the most important factor. Vinegar is a difficult product to taste, due to the intense sensations it provokes. The pungency of the high acetic acid content masks other flavors, and some familiarity with the product is required to proceed with a tasting. In fact, there is no consensus on how vinegar should be tasted. A vinegar sensory analysis panel requires well-trained tasters, and the specific attributes that are useful for differentiating among samples must be chosen.

To train a vinegar panel, Tesfaye et al. [34] used solutions of different concentrations of the compounds most typically found in wine vinegar, such as acetic acid, ethyl acetate, and wood extract obtained by maceration. The last two were prepared in 7% acetic acid to provoke a sensation similar to that of vinegar. Acetic acid aggressiveness determines the number of samples that can be examined in each session, and each sample is tasted four times. These four replicate samples should be tasted on different days to avoid sensorial saturation of the tasters. A descriptive analysis of the samples is prepared based on previously selected attributes that can be evaluated by the panel. The attributes used to describe the vinegar samples were color, aromatic intensity, woody scent, herbaceous smell, fruity odor of ethyl acetate, wine smell, and pungent feeling [34, 35]. Higher sensory thresholds for most compounds were obtained in an acetic acid matrix compared with water solutions. Conversely, high quality vinegars contain a large number of these compounds at concentrations higher than their threshold limits, including vanillin, eugenol, and benzaldehyde, and this characteristic could be therefore selected as an attribute of high quality vinegars [36]. Additionally, adequate training and a standardized tasting protocol contribute to the reliability of descriptive sensory analyses of a vinegar and of ascertaining the aging period and wood used in its elaboration [37].
